# Development of Cortical Lesion Volumes on Double Inversion Recovery MRI in Patients With Relapse-Onset Multiple Sclerosis

**DOI:** 10.3389/fneur.2019.00133

**Published:** 2019-02-22

**Authors:** Tobias D. Faizy, Gabriel Broocks, Christian Thaler, Geraldine Rauch, Pimrapat Gebert, Klarissa H. Stürner, Fabian Flottmann, Hannes Leischner, Helge C. Kniep, Jan-Patrick Stellmann, Christoph Heesen, Jens Fiehler, Susanne Gellißen, Uta Hanning

**Affiliations:** ^1^Department of Diagnostic and Interventional Neuroradiology, University Medical Center Hamburg-Eppendorf, Hamburg, Germany; ^2^Institute of Medical Biometry and Epidemiology, Charité Berlin-University Medical Center, Berlin, Germany; ^3^Department of Neurology, University Medical Center Hamburg-Eppendorf, Hamburg, Germany; ^4^Institute of Neuroimmunology and Multiple Sclerosis, University Medical Center Hamburg-Eppendorf, Hamburg, Germany; ^5^Department of Neurology, Christian-Albrechts University of Kiel, Kiel, Germany

**Keywords:** multiple sclerosis, cerebral cortex, inflammation, magnetic resonance imaging, cortical lesion volume, double inversion recovery

## Abstract

**Background and Objective:** In multiple sclerosis (MS) patients, Double Inversion Recovery (DIR) magnetic resonance imaging (MRI) can be used to detect cortical lesions (CL). While the quantity and distribution of CLs seems to be associated with patients' disease course, literature lacks frequent assessments of CL volumes (CL-V) in this context. We investigated the reliability of DIR for the longitudinal assessment of CL-V development with frequent follow-up MRIs and examined the course of CL-V progressions in relation to white-matter lesions (WML), contrast enhancing lesions (CEL) and clinical parameters in patients with Relapsing-Remitting Multiple Sclerosis (RRMS).

**Methods:** In this *post-hoc* analysis, image- and clinical data of a subset of 24 subjects that were part of a phase IIa clinical trial on the “**S**afety, Tolerability and Mechanisms of **A**ction of **B**oswellic **A**cids in Multiple Sclerosis (SABA)” (ClinicalTrials.gov, NCT01450124) were included. The study was divided in three phases (screening, treatment, study-end). All patients received 12 MRI follow-up-examinations (including DIR) during a 16-months period. CL-Vs were assessed for each patient on each follow-up MRI separately by two experienced neuroradiologists. Results of neurological screening tests, as well as other MRI parameters (WML number and volume and CELs) were included from the SABA investigation data.

**Results:** Inter-rater agreement regarding CL-V assessment over time was good-to-excellent (κ = 0.89). Mean intraobserver variability was 1.1%. In all patients, a total number of 218 CLs was found. Total CL-Vs of all patients increased during the 4 months of baseline screening followed by a continuous and significant decrease from month 5 until study-end (*p* < 0.001, Kendall'W = 0.413). A positive association between WML volumes and CL-Vs was observed during baseline screening. Decreased CL-V were associated with lower EDSS and also with improvements of SDMT- and SCRIPPS scores.

**Conclusion:** DIR MRI seems to be a reliable tool for the frequent assessment of CL-Vs. Overall CL-Vs decreased during the follow-up period and were associated with improvements of cognitive and disability status scores. Our results suggest the presence of short-term CL-V dynamics in RRMS patients and we presume that the laborious evaluation of lesion volumes may be worthwhile for future investigations.

**Clinical Trial Numbers:** www.ClinicalTrials.gov, “The SABA trial”; number: NCT01450124

## Introduction

Recent clinical trials, neuropathological investigations and MRI imaging studies have drawn growing attention to the relevance of gray matter (GM) lesions and particularly cortical lesions (CL) in Multiple Sclerosis (MS) patients (1–3). In contrast to earlier assumptions (4, 5), recent studies reported that neuroinflammation is present in CLs, suggesting that active immune mechanisms and focal demyelination are present inside the cortical layer (6–8). Histopathological reports revealed at least a partial independence in the evolution of CLs and white matter lesions (WML) (6, 9–11), suggesting different underlying neuroimmunological pathomechanisms regarding the development of both lesion-types. Moreover, CLs seem to correlate more closely with the degree of disability than the quantity of white matter lesions (WML) (2, 10, 12, 13). However, a comprehensive and frequent *in vivo* assessment of CLs is still challenging and little is known about structural developments of CLs in MS patients. Therefore, advanced imaging techniques, such as Double Inversion Recovery (DIR) MRI have shown considerable benefits for the evaluation of cortical lesion quantity and their distribution inside the cerebral cortex. DIR imaging has been shown to increase the detection rates of CLs inside the cerebral cortex and some comprehensive longitudinal investigations reported significant correlations of CL numbers (CL-N) with patients' disability status (1, 14, 15). While CL morphology remains unchanged over time (14), this may not seem to apply for CL-V (1, 14–16). However, current literature lacks longitudinal frequent assessments of CL-V on DIR in this context. In this study, we had access to a subset of longitudinal MRI data from the phase IIa trial on the “**S**afety, Tolerability and Mechanisms of **A**ction of **B**oswellic **A**cids (BA) in Multiple Sclerosis (SABA)” (17). This subset of RRMS patients underwent 12 MRI follow-up examinations (including DIR sequences) within a 16-months period enabling assessments of CLs at high sampling rates. The aim of this *post-hoc* study was 2-fold: (1) to investigate the reliability of DIR MRI for the assessment of CL-Vs and (2) to compare the course of CL-V progressions with those of other MRI biomarkers and clinical screening parameters during the follow-up period. We hypothesized that DIR MRI is a reliable tool for longitudinal assessments of CL-Vs and that the course of CL-V development is associated with clinical screening parameters, but independent from that of WMLs and contrast enhancing lesions (CEL), presumably due to their different underlying pathomechanisms.

## Methods

### Patients

This *post-hoc* study was approved by the local research Ethical Committee Hamburg (Ethik-Komission der Ärztekammer Hamburg) and the Federal Institute for Drugs and Medical Devices (BfArM), following the guidelines of the Declaration of Helsinki and written informed consent was given from every participant. In total, 24 relapsing-remitting MS (RRMS) patients, all diagnosed with definite MS based on the 2010 revised McDonald criteria (18) were enrolled. The study cohort was a subset of patients included in a phase IIa baseline-to-treatment clinical trial on boswellic acids (SABA) (17) to treat active RRMS (clinical trial number: NCT01450124). The inclusion criteria for this subset of SABA patients were as follows: (1) only patients with completed 16-month follow-up data were included (i.e., all clinical and demographic data were completely available and treatment maintained throughout the whole follow-up period); (2) the MRI investigation was conducted at the University Medical Center Hamburg-Eppendorf (data from other centers were excluded); (3) all included patients completed the full set of 12 DIR MRI. Baseline patients' characteristics are summarized in Table 1.

**Table 1:** Patients' baseline characteristics.

**Demographic data**	***n* = 24**
Age (years)-mean (SD)	38 (11)
Sex–female/male	20/4
EDSS (0–10 points)-median (IQR)	2.0 (1.5, 2.5)
Disease duration (years)-median (IQR)	7.4 (2.6, 11.5)

*EDSS, Expanded Disability Status Scale; Data are presented as mean or median values or absolute numbers as indicated*.

### Background Information on the SABA Study

SABA was designed as a multicenter baseline-to-treatment phase IIa trial on patients with RRMS and Clinically Isolated Syndrome (CIS) (17). The main study objective was to determine the safety and tolerability of high dose BAs in MS patients to describe the effect on disease activity. The trial was divided into 3 phases: Months 1–4 were considered as “screening phase” (SP), which delivered baseline disease activities without immunotherapy. Treatment with BAs was initiated at the beginning of month 5 and was maintained as long as the subject tolerated the drug. During months 5–7, patients were assessed for the tolerability and safety of the drug. The 4-months interval between 9 and 12 were regarded as “treatment phase” (TP), since noticeable therapy effects, i.e., visible on MRI images, were expected in that period. The end of the study was at month 16 and hence was declared as “study-end” (SE). Based on the SABA study regulations (17), MRI scans were conducted monthly, together with clinical screening tests, except for months 8 and 13–15. All patients were treated with BA doses up to 1,500 mg/3 times a day and received no corticosteroids or other immunomodulating substances for at least 3 months before entering the study.

### Clinical Scores

Results from patients' clinical neurological screening tests were taken from the main study data of SABA. Neurological screening included the Expanded-Disability-Status-Scale (EDSS) (19), the Paced Auditory Serial Addition Test (PASAT) (20) and the “Symbol Digit Modalities Test” (SDMT) (21). SDMT values are presented in relation to normative data from an age-matched and education-adapted normal population. Negative values represent a standard deviation below the age-matched and education-adapted normal population, indicating inferior test results of the patient and vice versa. Furthermore, the SCRIPPs neurological rating scale (22), as an additional disability measure, was acquired.

### MRI Image Acquisition

MRI examinations were conducted on a 3T MR scanner (Skyra, Siemens Medical Systems, Erlangen, Germany) with a 32-channel head coil. The MR protocol consisted of axial 2D T2w turbo spin echo (TSE) images acquired with TR = 2800 ms, TE = 90 ms, 43 slices, Matrix: 192 × 256, slice thickness = 3 mm and in plane resolution = 0.5 × 0.5 mm^2^, turbo factor = 5; 3D-FLAIR images with TR = 4700 ms, TI = 1800 ms, TE = 390 ms, 192 slices, slice thickness = 1 mm, matrix = 256 × 256; 3D DIR sequences were acquired with TR = 7500 ms, TE = 321 ms, TI1 = 3000 ms, TI2 = 450 ms, 192 slices, slice thickness = 1.4 mm, matrix = 256 × 256, in plane resolution = 1.4 × 1.4mm^2^; T1-MPRAGE pre and 5 min post Gadolinium with TR = 1900 ms, TE = 2.43 ms, TI = 900 ms, slice thickness = 1 mm, matrix = 256 × 256 × 192, voxel size = 1 × 1 × 1 mm and Gadolinium-dose of 0.2 ml per kilogram of body weight.

### Image Analysis

Image data were processed using the software package FSL 5.0 (Analysis Group, FMRIB, Oxford, UK). Lesion segmentation was performed with the software package Analyze 11.0 (Analyze Direct, Overland Park, KS, USA). All MRI examinations were assessed by two experienced neuroradiologists (T.F. and G.B.). CL identification was performed using a similar lesion scoring approach as described in (14). In brief: In a first reading step, CL-N and CL-V were assessed by each reader separately on correspondent DIR scans of each patient in random order. Hereafter, intraobserver and inter-rater reliability was computed. Intraobserver reproducibility was tested as described in (1). One observer (T.F.) evaluated all DIR scans of 24 MS patients 3×. The intraobserver variability was calculated by using the coefficient of variation, defined as the standard deviation (SD) of a random variable divided by its mean value (1). Secondly, consensus reading was performed and both readers judged on unclear or disputatious CLs together. Thirdly, each CL was numbered and traced throughout the follow-up of a patient by using a side-by-side comparison. Hereby, identified CLs were tracked as the same lesions in all follow-up MRI and new CLs were clearly identifiable as such during the time-course. CLs were manually selected and CL-Vs were delineated using a semi-automatic threshold-based region growing segmentation approach. Threshold-based lesion volumetry on DIR has successfully been utilized in related imaging studies (1, 14, 15). CL scoring was performed applying the “MAGNIMS consensus recommendations for MS cortical lesion scoring” (23). Consequently, two subtypes of CLs were differentiated, namely pure intracortical lesions and mixed gray/white matter lesions (consisting of leukocortical/juxtacortical lesions). For longitudinal CL-V analysis, both subtypes were considered, as proposed in the MAGNIMS consensus recommendations (24) and mean CL-V/follow-up visit (in mm3) for each patient was acquired. The number and volume (mm3) of WMLs and new WML/follow-up, the number of CELs, as well as new CEL/follow-up were taken from the SABA study data of each patient and included in our analysis.

### Statistical Analysis

Results were presented as mean (standard deviation, SD), median (25th percentile and 75th percentile) for continuous variables, depending on the distribution or frequencies with percentages for categorical variables. The change of CL-V and CL-N over a 16-month period were explored by using Friedman test and the effect size of Kendall'W was reported. A linear mixed model (LMM) was employed to describe the trend of CL-V over time and to assess the influence of MRI and clinical parameters. Hence, CL-V was set as dependent variable of the model with the time point of assessment in months as fixed factor. We explored the association of age, sex, and duration of disease, which was not significant. WML number and volume, CEL number, and the neurological screening parameters EDSS, SDMT, PASAT, and SCRIPPs were set as covariates of the model. A random intercept for the patient ID was included in the model to account for cluster correlation in the data. A first-order autoregressive structure with heterogeneous variances was assumed for the covariance structure. Interobserver reliability was assessed computing Cohen's κ. All statistical analyses were performed using SPSS software (IBM Corp. Released 2016. IBM SPSS Statistics for Windows, Version 24.0. Armonk, NY: IBM Corp.) and all graphics were created with Stata (Version 15SE; Stata Corp. 2017, College Station, TX). The significance level was considered as *p*-value ≤ 0.05 (two-sided).

## Results

### Reliability of Cortical Lesion Volume Assessment

Overall mean inter-rater agreement regarding CL-V assessment in all follow-up examination was good-to-excellent (κ = 0.89). Mean intraobserver variability was 1.1%.

### Cortical Lesion Volume Development

**Figure 1:**
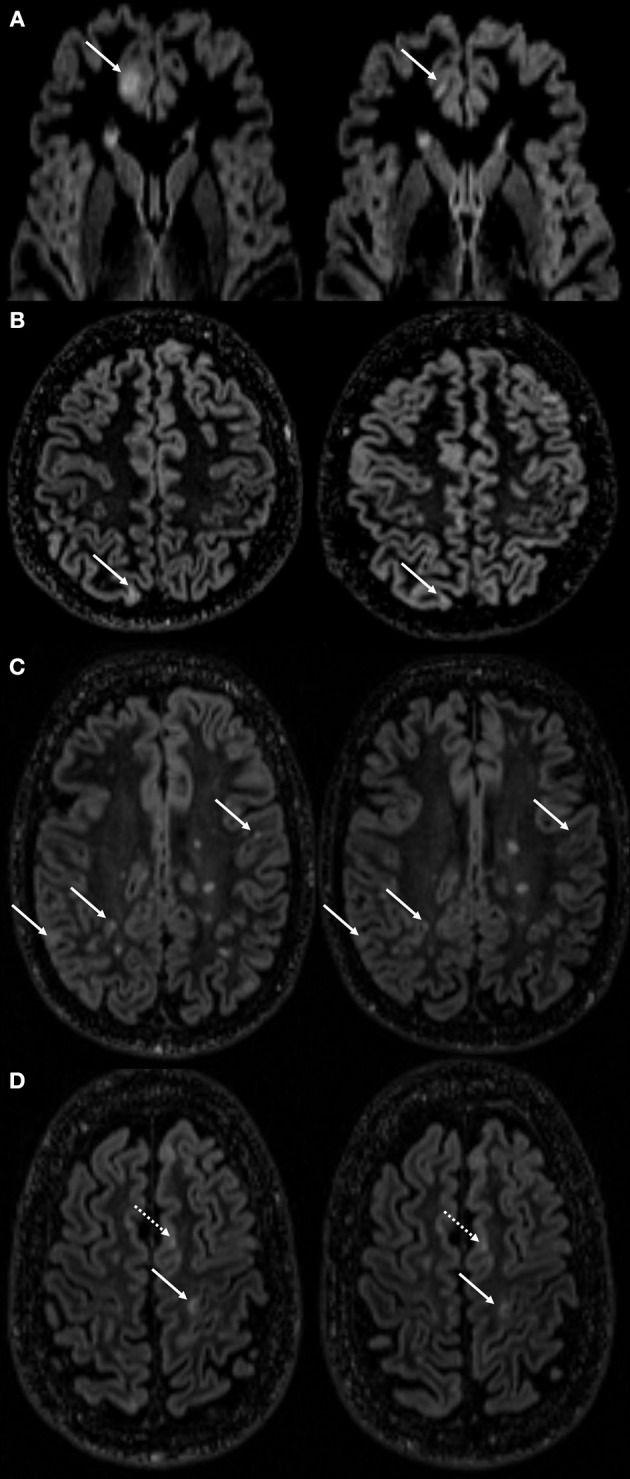
Examples of cortical lesion volume changes during follow-up. This figure displays cortical lesion volume (CL-V) changes of several lesions followed over the time course in different MS patients. Distinct volume reduction of a CL in the frontal cortex **(A)** marked with a white arrow at month 2 of screening phase (SP, left side) and month 3 of treatment phase (TP, right side). Rather marginal, but still notable CL-V reductions found in the cortices of patients in **(B)** (1st month of SP left and 2nd month of TP right) and **(C)** (2nd month of SP left and SE right). White arrow in **(D)** (4th month of SP left and 1st month of TP right) points at a cortical lesion with a measurable volume decrease. Dotted white arrow is indicating a CL with no measurable volume change.

**Figure 2:**
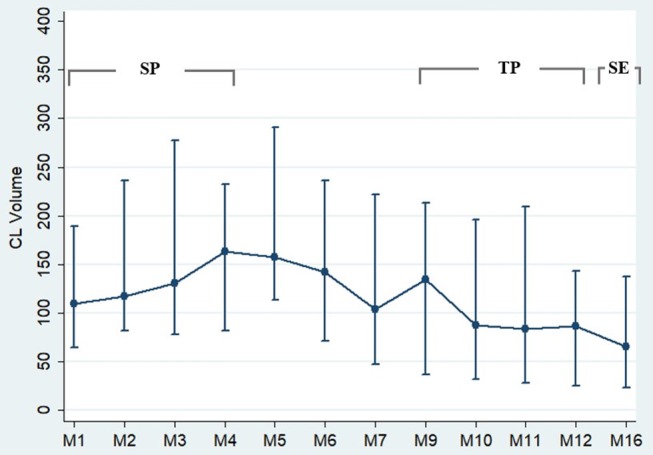
Cortical lesion volume progression during follow-up. This figure displays the progression of the cortical lesion volume (CL-V) during the time course. From month 1–5, CL-V is fluctuating with a tendency to an increase of volume. From month 5 onwards, a continuous decrease of CL-V was detected. *n* = 24. The circles represent the median values with the corresponding 25th percentile and 75th percentile.

Figure 1 displays examples of CL-V progressions and Figure 2 displays the development of median CL-V over time (by month), together with the corresponding 25th percentile and 75th percentile. Median CL-Vs increased during 4 months of baseline SP and then continuously and significantly decreased from month 5 until SE (*p* < 0.001, Kendall'W = 0.413). Summary measures (Supplementary Table 1) for median CL-Vs in all patients in the respective study phases were as follows: A median CL-V of 134.8 mm3 (7.3–1490) was detected during baseline SP. During TP, the median CL-V decreased to 91.7 mm3 (3.7–1239.6). At SE, a median CL-V of 65.1 mm3 (0–995.7) was measured. LMM analysis was performed to evaluate associations of the course of CL-V development and other MRI biomarkers. During baseline SP, a significant relationship between CL-V and WML volume (*p* = 0.03) and EDSS (*p* = 0.04) was detected (not displayed). To further explore the factors associated with a decrease of CL-V after baseline SP, LMM analysis between months 5 to 16 was performed (Table 2). In this period, lower CL-Vs were associated with lower EDSS scores (*p* = 0.05) and higher SDMT (*p* = 0.048) and SCRIPPs scores (*p* = 0.006). No significant association was found between CL-V and WML number (*p* = 0.526) or volume (*p* = 0.881) or CELs (*p* = 0.287).

**Table 2:** Cortical lesion volume progression in relationship with MRI and clinical parameters.

**Factors**	**Unstandardized coefficient**	**95% CI of unstandardized coefficient**	***p*-value**
Intercept	−268.91	−514.63 to −23.20	0.032^*^
Time (months)	−7.39	−10.46 to −4.32	0.002^*^
SCRIPPs	−3.29	−5.59 to −0.99	0.006^*^
PASAT	0.57	−0.35 to 1.49	0.211
SDMT	−6.38	−16.38 to −3.62	0.048^*^
EDSS	12.64	0.02 to 25.30	0.050^*^
WML volume	−0.0001	−0.004 to 0.004	0.881
Number of WML	−0.507	−2.09 to 1.07	0.526
Number of CEL	1.89	−1.65 to 5.43	0.287

*This table displays the results of the general linear model regarding the progression of Cortical Lesion Volume (CL-V) from month 5 (treatment onset) to month 16 (study end). Since months 1–4 showed no significant trend in CL-V dynamics, this period was not included in this analysis. The dependent variable in this model was CL-V. WML, white matter lesion; CEL, contrast enhancing lesions; EDSS, Expanded Status Disability Scale; SDMT, Normative Symbol Digit Modalities Test; PASAT, Paced Auditory Serial Addition Test (out of 60 possible answers); SCRIPPs, score of disability measurement; significant values (p ≦ 0.05) are marked by an asterisk*.

### Cortical Lesion Quantification

**Figure 3:**
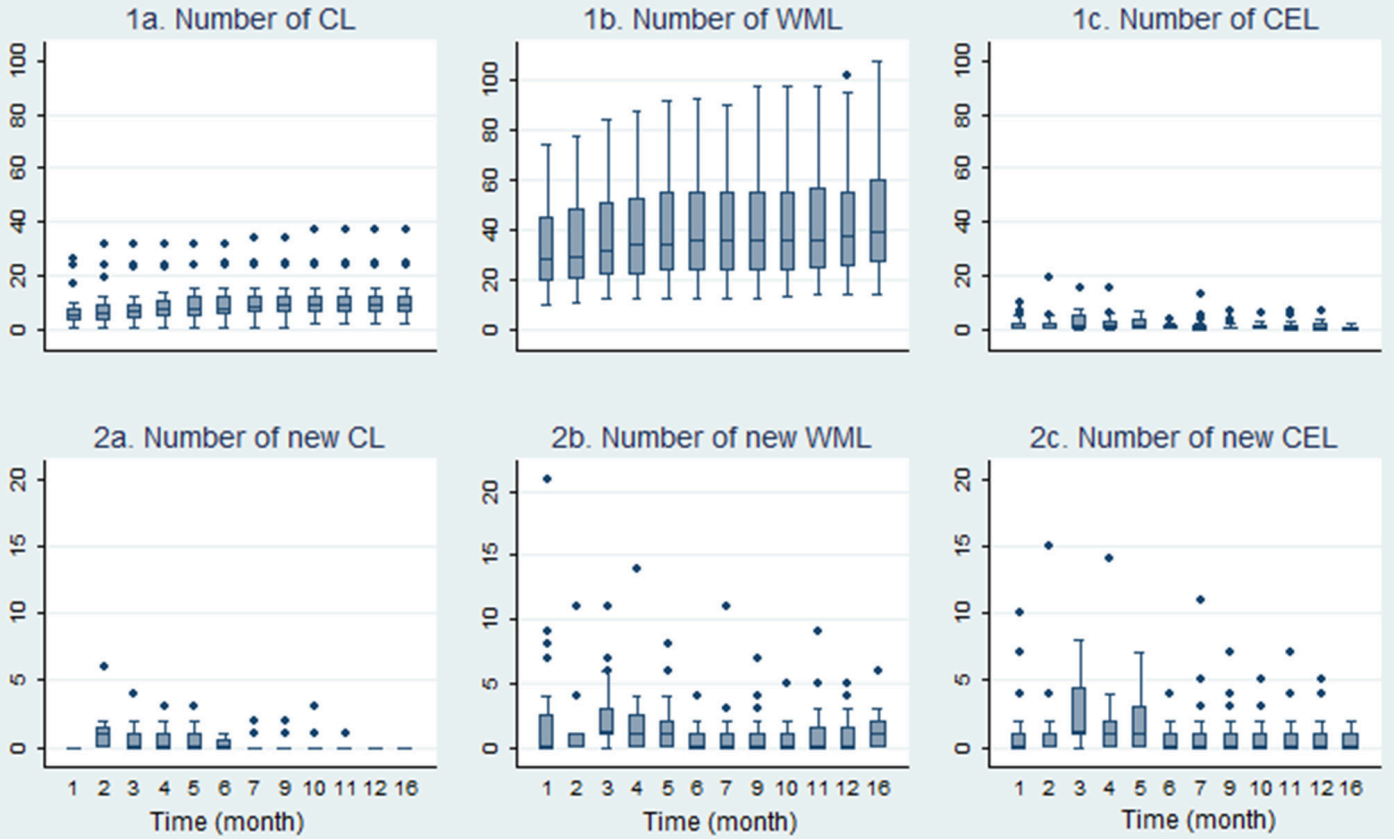
Development of the course of cortical-, white-matter- and contrast enhancing lesions. Boxplots in this figure display the development of lesion numbers and the number of new lesions during the 16-month observation period (*n* = 24). CL, cortical lesion; WML, white matter lesion; CEL, contrast enhancing lesion.

A total number of 218 CLs were found by consensus. At baseline scan, at least one CL was found in 23/24 patients. One hundred and Sixty one CLs (~ 74%) were considered as mixed gray/white matter lesions (leukocortical lesions/juxtacortical lesions), whereas 57 CLs (~26%) were regarded as pure intracortical lesions. We detected a significant increase of total CL-Ns (*p* < 0.001, Kendall'W = 0.748), but fewer new CLs/follow-up emerged (*p* < 0.001, Kendall'W = 0.227) during the 16-months observation period (Figure 3). Supplementary Table 1 displays summary measures of (new) CLs in the related study-phases.

### Contrast Enhancing Lesions

In accordance with findings of the SABA study, a reduction of CELs during the observation period was detected (Figure 3). There was a statistically significant decrease of CEL numbers (*p* < 0.001, Kendall'W = 0.180) and new CEL/follow-up (*p* < 0.001, Kendall'W = 0.154) during follow-up (Figure 3).

### T2w White Matter Lesion Numbers and Volumes

Summary median WML values are displayed in Supplementary Table 1. During the 16-months observation period, the total number of WML lesions (*p* < 0.001, Kendall'W = 0.832) and new WML/follow-up (*p* = 0.008, Kendall'W = 0.097) increased significantly. Figure 3 depicts the course of median WML per follow-up month.

## Discussion

CL-V development has been assumed to be independent from WML evolution, but seems to be associated with the progression of patients' disability status and clinical course (10, 14, 25). Therefore, standardized assessments of CL-Vs may be worthwhile and of potential to be used to optimize patient care in clinical routine. Advanced MRI techniques, such as DIR, may not only reveal the quantity of CLs located in the cerebral cortex, but may also be suitable for CL-V assessment (26). However, longitudinal, high sampling rate evaluations of CL-V development on DIR are scarce and literature yet lacks a comprehensive investigation with patients incorporated in a standardized imaging and treatment protocol. In this *post-hoc* analysis, we aimed to frequently assess the development of CL-Vs in a subset of RRMS patients from the SABA trial (17). We observed small, but notable short-term CL-V dynamics in all included patients. Overall CL-Vs did not change significantly during the 4 months of SP, while a decrease of CL-V was evident during follow-up. Intrarater variability regarding CL-V assessment on DIR was comparably small (1.1%) and overall inter-observer agreement on DIR images was good-to-excellent. We conclude that DIR MRI is a reliable tool for CL-V assessments and presume that the evaluation of CL-Vs may hold promising potentials for comprehensive future clinical-radiological investigations in MS patients.

CLs in general and, with restrictions, CL-Vs have been evaluated in some recent longitudinal DIR MRI studies incorporating MS patients. However, most of these investigations conducted only very few follow-up timepoints, restricting their ability to determine the reliability of DIR for the assessment of CL-V (1, 14, 15). However, unanimously, many recent studies reported of *de-novo* formations of CLs even within short time intervals, which seem to be correlated with the patients' individual disability status 13, 14, 10, 27, 16]. In contrast, only very few comparable imaging studies exist, which analyzed inter-rater reliabilities or intraobserver variances regarding the detection rates of CLs (and CL-Vs) (1, 14, 15). However, the intraobserver variance and inter-rater reliability detected in our investigation was slightly better compared to the referred studies. One explanation may be that the overall average CL-Vs detected in our cohort were larger compared to the related studies. This presumption is supported by other imaging studies, which reported that lesions with greater lesion volumes (on DIR and other MRI sequences) may be detected more reliably by the investigators (14, 16, 27). Our results suggest that DIR MRI is a reliable tool for the frequent assessment of CL-Vs in MS patients.

Comparable longitudinal studies reported of discordant associations of CL-Vs with clinical parameters. A 3-year longitudinal study by Calabrese et al. (1) including 76 RRMS patients reported of a significant increase of CL-V especially in RRMS patients with a clinical worsening reflected by EDSS changes. Moreover, CL-V at baseline scans correlated significantly with WML-volume, disease duration and EDSS. The authors stated that CL-V predicted disability accumulation over the study period. Another study by Calabrese et al. (14) also assessed CL-V changes in a cohort of RRMS and SPMS patients during a time space of 1-year. The authors did not find any significant changes in CL-V after 1-year compared to baseline measures, while CL-N increased during follow-up. A study by Mike et al. (27), though not longitudinal in nature, reported of correlations of CL-V with WML volume and CL-N, but also with SDMT and EDSS measures. Furthermore, the authors found a trend toward higher CL-V with disease duration and progression when comparing early RRMS vs. late RRMS patients. These results are in accordance with our findings, since we also detected an association between CL-Vs and EDSS, SDMT and SCRIPPs scores during follow-up. CL-V showed a relationship with WML volume during SP, but was not associated with either the course of WML number or volume, or parameters of disease activity (CEL) after treatment initiation. This finding supports the claim of an at least partial independence of CL-V development from parameters of WM inflammation, which was raised in previous reports (1, 6, 8, 15). However, all these findings support the assumption that (the magnitude of) CL-V indeed may be another useful biomarker related with patients' disability status and clinical course, justifying the laborious evaluation of these lesions. Nevertheless, although the assessability of CLs has clearly been improved by advanced imaging techniques such as DIR or Phase-Sensitive Inversion Recovery (26, 28), lesion identification is still challenging (10, 16, 23) and time-consuming and therefore may not be easily utilized outside of a research environment. For the future, automated lesion identification tools or machine learning algorithms under supervision of an experienced neuroradiologist may enable fast and reliable CL-N and CL-V quantifications, which may pave the way for the evolution of CL dynamics in the clinical routine. Notably, promising results in this field have just recently been reported for automated WML detection approaches (29).

While many investigations focused on microstructural and cellular composition of CLs (5, 6, 8, 10, 13, 30, 31), little is known about the underlying pathophysiology of CL-V changes. In contrast to the assumption that specific widespread histo- and immuno-pathological patterns of focal inflammation, which are evident in WMLs, are missing in case of cortical demyelination (4, 5, 8), current data suggest that CLs indeed hold extensive amounts of inflammatory components (6–8). Histopathologically, the majority of CLs contain T-cell infiltrates, B-cell follicle-like-structures, as well as conglomerates of microglia, lymphocytes and macrophages, suggesting a direct role of these inflammatory cells in cortical inflammation (6). Interestingly, mixed gray/white matter lesions (which represent most CLs detected in our study) seem to be more inflammatory than subpial or pure intracortical lesions (6). It may be conceivable, albeit speculative, that CL-V sizes are connected to the magnitude of cortical inflammation and thus may be targeted by anti-inflammatory treatments. Whether a decline of CL-V may appear due to a reduction of cellular inflammatory components or due to an anti-oedematous effect needs to be objective of further investigations including a control group. As for now, it is unclear if our findings display the natural history of the disease (with improvement or resolution of acute flare) or any kind of treatment response. Future longitudinal investigations including greater amounts of subjects, histopathological specimens and different treatment regimens may reveal the implications of CL-V progressions for patients' individual disease course, degree of cortical inflammation and relapse status.

Our study holds some limitations: Due to the high frequency of almost monthly follow-ups, a “learning-effect” to the clinical screening tests, especially for the evaluation of neurocognitive functioning is evident. Therefore, no valid claims regarding associations between neurocognitive measures and CL-V evolution can be made. Our study model intrinsically does not hold a control group. Any claims about treatment effects of BAs are not scope of the manuscript. The follow-up period of this study is relatively short. Long-term alterations of CL-Vs need to be investigated in more extended longitudinal studies. DIR sequences are prone to artifacts and suffer from a comparably low signal-to-noise ratio. It has been reported, that only a minority of CLs are actually visible on DIR scans and that the classification of distinct CL types can be challenging (10, 16). Therefore, we used a two-reader consensus approach to minimize misinterpretations of CL-N and CL-V accordingly. However, we cannot exclude that in some cases, due to the technical limitation of the sequence, CL-Vs may be slightly over- or under-estimated.

## Conclusion

DIR MRI seems to be a reliable tool for the assessment of CL-V development over time. CL-V decreased evidently during follow-up, indicating short-term structural and quantitative lesion dynamics in RRMS. CL-V was not associated with the course of WML number and volume or CELs, supporting the assumption of different pathophysiological (inflammatory) mechanisms affecting the emergence and the progression of these biomarkers. We conclude that the laborious evaluation of CL-Vs may be worthwhile and should be further investigated in future clinical studies including healthy control groups.

## Data Availability

The raw data supporting the conclusions of this manuscript will be made available by the authors, without undue reservation, to any qualified researcher.

## Author Contributions

TF, SG, JF, CT, GB, J-PS, CH, KS, UH: Conception and design of the study. TF, SG, GB, CT, FF, GR, PG, HK, KS, HL: Acquisition and analysis of the data. TF, GB, JF, CH, J-PS, HK, UH: Drafting a significant portion of the manuscript or figures. PG, GR: Professional Statistical Support.

### Conflict of Interest Statement

The authors declare that the research was conducted in the absence of any commercial or financial relationships that could be construed as a potential conflict of interest.

## Abbreviations

CEL: Contrast enhancing lesions
CL: Cortical lesions
CL-V: Cortical lesion volume
DIR: Double Inversion Recovery
PASAT: Paced Auditory Serial Addition Test
RRMS: Relapsing-remitting Multiple Sclerosis
SDMT: Symbol Digit Modalities Test
WML: White matter lesion.
