# Supplementary material for: Development of Cortical Lesion Volumes on Double Inversion Recovery MRI in Patients With Relapse-Onset Multiple Sclerosis
Source: Front Neurol. 2019 Feb 22;10:133. doi: 10.3389/fneur.2019.00133 (PMC6401630; doi:10.3389/fneur.2019.00133)
Supplement: Supplementary file 1 [file Table_1.DOCX]

Supplementary Material

# Supplementary Table 1

**MRI findings at the different study phases (SP, TP, SE)**

| Parameter | Screening phase (SP) | Treatment phase (TP) | Study End (SE) |
| --- | --- | --- | --- |
| CL volume [mm³] | 134.78 (7.34-1490.87) | 91.69 (3.67-1239.63) | 65.10 (0-995.74) |
| WML volume [mm³] | 9003.50 (175-27375) | 8826.50 (1595-27634) | 8834 (1595-22979) |
| Number of CL | 6 (1-32) | 7 (1-37) | 7 (2-37) |
| New CL / follow-up | 0 (0-6) | 0 (0-3) | 0 (0-1) |
| Number of WML | 31.50 (3-88) | 36 (12-102) | 39 (14-108) |
| New WML / follow-up | 4 (0-21) | 0 (0-9) | 1 (0-6) |
| Number of CEL | 2 (2-19) | 0 (0-7) | 0 (0-5) |
| New CEL / follow-up | 1 (0-15) | 0 (0-7) | 0 (0-5) |

Legend: CEL = contrast enhancing lesion; CL = cortical lesion; WML= white matter lesion; Summary measures for each study phase are displayed as median and (range). SP and TP each consisted of 4 follow-up visitations (1 per month) and SE consisted of 1 final follow-up examination.
